# Hospital Meetings, &c

**Published:** 1897-03-13

**Authors:** 


### HOSPITAL MEETINGS, &C.

### WEST HAM HOSPITAL.

The annual meeting of the governors and subscribers of the West Ham Hospital was held in the Dispensary Hall on Wednesday, 24th ult., under the presidency of Mr. G. Hay. 580 in-patients were admitted during the year 1896 as against 441 in the previous twelve months, an increase of 139. In the out-patient department the cases numbered 21,924, of whom 10,667 were admitted by letter of recommendation; 8,664 were casualties and cases of emergency; and 2,593 were dental cases. The committee again called attention to the Adult Samaritan Fund, which had not benefited by a single contribution during the past two years. The Children's Samaritan Fund has been instrumental in sending children to seaside convalescent homes to aid their restoration to health. The ordinary receipts of the hospital amounted to £3,385 8s. 9d. as against £5,118 11s. 4d. in the preceding twelve months. This decrease is attributed to the diminution in donations and workpeople's contributions. £270 in legacies were also received. The ordinary expenditure was £4,043, in addition to which £42 was spent on extraordinary repairs, &c. On the motion of the Chairman, seconded by Mr. F. Smith, the report was adopted. The committee of management was re-elected, and the meeting concluded with votes of thanks to the officers and the chairman.

### NATIONAL ORTHOPÆDIC HOSPITAL.

The Duke of Marlborough presided at the annual meeting of the National Orthopædic Hospital which was held at the hospital on Friday, 26th ult.

The report, which was adopted on the motion of the Chairman, seconded by the Rev. A. J. Robinson, stated that 128 children and 99 adults had received treatment as in-patients during the year, and 1,238 out-patients had been entered on the books. The income (including a legacy of £100) amounted to £2,269 and the expenditure to £2,280.

The remainder of the proceedings consisted of routine business.

### ROYAL HOSPITAL, RICHMOND.

The Duke of Teck presided on Saturday, 27th ult., over the annual meeting of the Royal Hospital, Richmond. The report stated that the year 1896 had been one of the most eventful in the history of the institution, and since its opening the cots in the Princess May's Ward had been constantly filled. The action of the governors in deciding to make this addition to the hospital had, therefore, been fully justified. Alluding to the growth of the hospital during the past year and the assistance rendered by religious bodies in the town, the Chairman said he trusted that still greater interest would be taken in the hospital during the present year. It was stated at the meeting that a movement had been instituted to permanently endow the hospital. Already £2,000 had been secured, and it was hoped that £10,000 would be obtained before the celebration of the Queen's reign in June.

### LONDON ORPHAN ASYLUM.

The eighty-fourth anniversary dinner of the London Orphan Asylum was held at the Hotel Metropole on Monday, 1st inst., the Earl of Verulam presiding. After the usual loyal toasts, the Chairman, in proposing "Success to the London Orphan Asylum," said that among other things this charity possessed a house for 50 boys which was subscribed for by generous donors in Hertfordshire, called the Hertfordshire House. The orphans taken into the institution, however, were not inhabitants of Hertfordshire alone, nor yet from the metropolis alone, as the name would seem to imply, but they were admitted from every part of the British Empire. Five hundred British boys and girls were at the present time members of the institution, and when his hearers reflected that the number when the institution was founded, was only five, they would see what a great advance had been made. Since the foundation, 5,947 children had been benefited, and £851,600 had been spent in their maintenance, education, and clothing. In January last 40 members were elected to the institution and 60 were unsuccessful. They wished in this, the sixtieth year of Her Majesty's reign, to make a special effort to elect 60 children—one for each year of that reign—in June next. Nothing, in his opinion, could be more appropriate than this, and he looked to his hearers in their generosity to contribute the necessary funds. The Treasurer (Mr. A. C. C. Arbuthnot), in responding to his own health, said that they had taken a great responsibility on their shoulders in admitting 60 children next June, as it would involve in the end an expenditure of some £10,000. A collection amounting to £3,220 was made as the result of the dinner. The chief items were the Chairman's list, £1,050; the Stock Exchange list, £1,294; and the ex-scholars' list (including 150 guineas from the L.O.A. Club), £184.

### ROYAL HOSPITAL FOR CHILDREN AND WOMEN.

The Lord Mayor and the Sheriffs attended in State at the Royal Hospital for Children and Women on Thursday, 4th inst. After proceeding through the wards, the Lord Mayor, who was accompanied by the Lady Mayoress, took the chair at the annual meeting, which was held in the Alexandra Ward.—The Rev. F. C. Bainbridge Bell, the chaplain of the hospital, moved the adoption of the report. This was seconded and carried.—The Lord Mayor, in moving a resolution, expressing confidence in the hospital, and opening a subscription list for the purpose of collecting £10,000 as a Diamond Jubilee gift for the hospital, made a stirring appeal on behalf of the institution.—Mr. J. Roles Hoare, in seconding, complained of the lack of hospital accommodation in London south of the Thames, when compared with that on the north side of the Thames. Out of a total population of about 4,232,000 there were about 2,687,000 persons in North London, and about one and a half millions in South London; while the number of hospital beds actually provided was 3,975 and 986 respectively. That was to say that the south side of the Thames, on a population basis, required 1,300 more beds to put them on a level with the north side. In another matter, too, he considered the south side was hardly treated, and that was in the distribution of the Hospital Sunday Fund. In 1895, out of the £53,000 distributed, South London, on a basis of population, ought to have received £16,700, whereas they (the poorer side of the metropolis) only received about £4,000, although they collected in their poor churches over £10,500 for the fund.

The treasurer, chairman, and other officers of the institution were re-elected, and votes of thanks brought the meeting to a close.

### CHARING CROSS HOSPITAL.

The Lord Mayor, who was accompanied by the Lady Mayoress, presided over a meeting at the Mansion House on Friday, 5th inst., in aid of the Special Fund Appeal of the Charing Cross Hospital.

The Lord Mayor having briefly opened the proceedings,

The Bishop of London proposed the following resolution:—

"That this meeting fully recognises the great services rendered to the public by Charing Cross Hospital during the 75 years of its existence, and emphatically declares its opinion that the hospital should be generously supported and maintained in its unique position."

The threat with which the appeal which had been issued on this occasion concluded, viz., that unless there was a generous response the hospital could not be carried on, was one which no one could contemplate with equanimity. The carrying out of such a threat would be almost a national disaster.

Lord Kinnaird seconded the resolution, which was carried.

The Hon. W. F. D. Smith, M.P., moved—

"That the usefulness of the Hospital is seriously crippled in its work by want of adequate accommodation and that additional buildings are urgently necessary to meet the want."

Dr. F. Henry Green seconded, and the resolution was carried.

Mr. Albert G. Sandeman, Governor of the Bank of England, proposed—

"That for the purpose of maintaining this Hospital, and extending its usefulness, it is imperative that a special appeal should be made, and this meeting pledges itself to use every effort on behalf of the appeal for £100,000 to be raised in commemoration of Her Majesty's reign."

The Rev. Prebendary J. F. Kitto, in seconding, said it was not possible to state the case for the hospital too strongly. Unless the hospital got the support it now wanted it could not go on.

The resolution was carried.

A vote of thanks to the chairman, proposed by Mr. T. P. Borrett, seconded by Mr. J. Astley Bloxam, concluded the proceedings.

It was announced that the total received up to the day of the meeting by the Special Appeal Committee was £17,696.

### ROYAL WESTMINSTER OPHTHALMIC HOSPITAL.

The annual meeting of the Royal Westminster Ophthalmic Hospital was held at the Hospital on Friday, 5th inst., Sir Charles A. Turner, K.C.I.E. (chairman of the committee), presiding. The Chairman proposed the adoption of the report, which stated that 558 in-patients had been treated in 1896, as compared with 468 in the previous year. 10,301 new out-patients also attended. The committe appealed for funds to enlarge and improve the building so as to provide additional and improved accommodation for the patients, the students, and the nursing staff. At the end of the year the building fund amounted to £1,714. The general income (including £250 received in legacies) had amounted to £2,301, and the expenditure (including £151 exceptional repairs) to £2,386. The Hon. Arthur Broderick seconded, and the report was adopted. The committee and hon. medical staff having been elected, a vote of thanks to the chairman brought the meeting to a close.

### THE NEW HOSPITAL FOR WOMEN.

Mrs. Creighton presided over the annual meeting of the New Hospital for Women, which was held at the hospital on March 4th. Mrs. Garrett Anderson, who moved the adoption of the report, drew special attention to a fact which certainly reflects the utmost credit upon the medical and surgical staff, *i.e.*, the extremely small mortality. The total number of deaths for the year was only eight, of which one only was a surgical case. Yet there were no less than 74 major and 192 minor operations during the year. Mrs. Anderson, commenting on the splendid results of modern surgery, told how much she had been struck by certain words of Sir James Paget, in days before Lister's discoveries were known, when he had said that the surgeons of the future must spend their lives in "keeping out mischief."

The Rev. Luke Paget, Mrs. Scharlieb, and Dr. Cullingworth were amongst other speakers. Mrs. Scharlieb alluded to a generous offer lately made to the hospital by the Rev. James Chadburn, to partially endow five beds for patients suffering from cancer, and suggested that his was an example which might well be followed by other people in this rich City of London. Commenting on the fact that for the first time the hospital had incurred a small debt, Mrs. Scharlieb said that she feared that because they at the New Hospital for Women had been honest and careful, they would suffer for it and would be supposed to require little help. Comparing the expenditure of the hospital with that of another of the same size, and of the same character, the latter had, said Mrs. Scharlieb, spent £1,500 more during the year.

Dr. Cullingworth, who moved a vote of thanks to the medical and nursing staffs, and the secretary, congratulated the medical staff on the remarkable results shown in the report of the year's work, results which showed that the members of the staff were "saturated with the antiseptic idea." He spoke of the value of the hospital as a teaching institution, adding that the wards reflected the greatest credit on the nursing staff.

After the meeting Miss Cartwright dispensed tea to the visitors, and many took the opportunity of going into the wards and admiring their bright and cheery aspect for themselves.

### METROPOLITAN HOSPITAL.

The annual meeting of the Governors of the Metropolitan Hospital was held at the hospital on Monday, 8th inst., the Chairman (Mr. C. J. Thomas) presiding. In moving the adoption of the report the Chairman referred to the great loss the hospital had sustained by the death of the late Chairman, Mr. Joseph Fry, one of the founders of the hospital. From the report we note that the number of patients treated showed an increase—in the case of in-patients of 42 (1013 in 1896 and 971 in 1895), and in the case of out-patients of 5,428 (26,768 and 21,330 respectively). The ordinary income was £1,905 less than the ordinary expenditure, but with the legacies received (£2,866) there is, as far as the year 1896 is concerned, a small balance in favour of the hospital. The hospital, however, is in debt to the tune of £9,500. Mr. J. R. Pike seconded, and the report was adopted. The retiring members of the committee and the auditors were re-elected, and a vote of thanks to the chairman concluded the meeting.

### ST. PETER'S HOSPITAL.

The annual meeting of the governors and subscribers to the St. Peter's Hospital for Stone was held at the hospital on Thursday, 4th inst., the Treasurer (Mr. F. A. Bevan) in the chair. During the year 1896 474 in and 4,799 new out patients, the largest number ever recorded, were treated. According to the report, the cost per in-patient per week had decreased from £2 9s. 3d. in 1895 to £2 5s. 9d. The total ordinary income amounted to £3,660, of which £2,237 was contributed by the patients. In addition, a legacy of £47 was received. The ordinary expenditure amounted to £3,354, and the extraordinary expenditure to £162.

### LONDON TEMPERANCE HOSPITAL.

The annual public meeting of the supporters of the London Temperance Hospital was held on Monday, 8th inst., at the hospital in Hampstead Road. The Duke of Westminster, president of the hospital, occupied the chair. The meeting was well attended, amongst those present being the Duchess of Westminster, the Archbishop of Canterbury and Mrs. Temple, Sir Wilfrid and Lady Lawson, Sir John and Lady Hutton, and the Hon. Conrad Dillon. Dr. Dawson Burns the hon. secretary, in giving an abstract of the annual report, said that last year there were 1,157 in-patients, and in only one case was it deemed advisable to administer alcohol, and there had not been more than twenty such cases during the whole existence of the hospital. The Duke of Westminster commended the appeal of the board of management for the immediate raising of a special fund of £10,000. The Archbishop of Canterbury moved, "That this meeting receives with great and unqualified satisfaction the statements submitted concerning the progress and success of the London Temperance Hospital, and warmly sympathises with the desire of the board of management that the proposed special fund of £10,000 should be speedily subscribed. This meeting also, in rejoicing that the Prince of Wales's Fund for the benefit of the London hospitals has been so favourably welcomed, earnestly trusts that this excellent endeavour to supplement existing means of support will not occasion any diminution of the public contributions which have hitherto been placed at the disposal of their managing board." Sir Wilfrid Lawson, M.P., seconded the motion, which was unanimously carried.

